# Melatonin Plays a Critical Protective Role in Nicotine-Related Abdominal Aortic Aneurysm

**DOI:** 10.3389/fphys.2020.00866

**Published:** 2020-07-17

**Authors:** Liren Duan, Shenli Li, Lei Wang, Yuchen Jing, Guangxin Li, Yaodong Sun, Weifeng Sun, Yalun Li, Lin Zhao, Shijie Xin

**Affiliations:** ^1^Department of Vascular Surgery, The First Hospital of China Medical University, Key Laboratory of Pathogenesis, Prevention and Therapeutics of Aortic Aneurysm, Shenyang, China; ^2^Department of Anesthesiology, The People’s Hospital of Liaoning Province, Shenyang, China; ^3^Department of Surgery, Yale University School of Medicine, New Haven, CT, United States; ^4^Department of Anorectal Surgery, The First Hospital of China Medical University, Shenyang, China; ^5^Department of Pharmacology, School of Pharmacy, Liaoning Key Laboratory of Molecular Targeted Anti-Tumor Drug Development and Evaluation, Shenyang, China

**Keywords:** melatonin, nicotine, abdominal aortic aneurysm, vascular smooth muscle cell, phenotypic switch

## Abstract

**Aim**: Smoking is a major risk factor for abdominal aortic aneurysm (AAA). Among the components of smoke, nicotine is known to exert pro-atherosclerotic, prothrombotic, and proangiogenic effects on vascular smooth muscle cells (VSMCs). The current study was designed to investigate the mechanisms through which nicotine induces vascular wall dysfunction and to examine whether melatonin protects against nicotine-related AAA.

**Methods**: In this study, an enzyme-linked immunosorbent assay (ELISA) was used to measure melatonin and TNF-α levels, as well as total antioxidant status (TAS), in patients with AAA. We established a nicotine-related AAA model and explored the mechanisms underlying the therapeutic effects of melatonin. Tissue histopathology was used to assess vascular function, while western blotting (WB) and immunofluorescence staining were performed to detect protein expression.

**Results**: We observed melatonin insufficiency in the serum from patients with AAA, particularly smokers. Moreover, melatonin level was positively correlated with antioxidant capacity. In the *in vivo* model, nicotine accelerated AAA expansion and destroyed vascular structure. Furthermore, OPN, LC3II, p62, matrix metalloproteinase-2 (MMP-2), matrix metalloproteinase-9 (MMP-9), NF-κB p65, TNF-α, phosphorylated AKT, and phosphorylated mTOR levels were increased, *in vivo*, following nicotine treatment, while SM22α and α-SMA levels were reduced. Additionally, melatonin attenuated the effects of nicotine on AAA and reversed changes in protein expression. Moreover, melatonin lost its protective effects following bafilomycin A1-mediated inhibition of autophagy.

**Conclusion**: Based on our data, melatonin exerts a beneficial effect on rats with nicotine-related AAA by downregulating the AKT-mTOR signaling pathway, improving autophagy dysfunction, and restoring the VSMC phenotype.

## Introduction

Abdominal aortic aneurysm (AAA) is a life-threatening disease. Aneurysms are progressive, constant, and degenerative, ultimately ending in rupture (Erbel et al., 2014). Among people older than 65 years, the incidence of AAA is greater than 5%, with death likely, should the aneurysm rupture (Brewster et al., 2003; Mitchell et al., 2010). Currently, open surgical repair or endovascular aneurysm repair (EVAR) is the main method through which AAAs, with a diameter larger than 50 mm, are treated. However, for early AAAs, surgical interventions do not achieve expected benefits, and no effective drugs are currently available. Therefore, drug therapy is still required in the early stage of AAA.

Although the causes of AAA remain unclear, smoking is an important risk factor for its development (Nordon et al., 2011; Lindquist Liljeqvist et al., 2017). Smoking increases the risk of AAA by 15-fold in males (Wong et al., 2007) and has increased the overall incidence of AAA by 4% annually (Wilmink et al., 1999). A potentially more concerning issue is that smoking accelerates AAA expansion and increases the risk of rupture (Kent et al., 2010; Li et al., 2018). Nicotine is the main pharmacological component of smoke and may affect the cardiovascular system by acting on vascular smooth muscle cells (VSMCs).

Melatonin, also known as N-acetyl-5-methoxytryptamine, is an indole hormone secreted by the pineal gland, which mainly functions to regulate circadian rhythm. In recent years, melatonin has been reported to play an important role in regulating the function of various organs, particularly in individuals with cardiovascular diseases such as hypertension, ischemic heart disease (IHD), and atherosclerosis (Sun et al., 2016). Additionally, endogenous melatonin levels are associated with a variety of cardiovascular diseases, such as myocardial infarction (Dominguez-Rodriguez et al., 2008). Furthermore, melatonin inhibits matrix metalloproteinase (MMP) activation and significantly inhibits the NF-κB signaling pathway, which is essential for the treatment of AAA (Tai et al., 2010; Chang et al., 2012; Shi et al., 2012; Rudra et al., 2013). Interestingly, melatonin alone has been shown to reverse AAA expansion (Kong et al., 2017; Tekin et al., 2018); however, an association between endogenous melatonin levels and AAA has not been observed, with the effect of melatonin on nicotine-related AAA still unclear. In the present study, we report an intrinsic relationship between melatonin and patients with AAA and attempt to explore melatonin’s therapeutic effects and potential mechanisms of action using a nicotine-related AAA model. Our findings may provide insights into the development of new drug treatments for AAA.

## Materials and Methods

### Clinical Data

We selected 100 patients diagnosed with AAA, based on the results of a computed tomography (CT) examination at the Department of Vascular Surgery in The First Affiliated Hospital of China Medical University. Measured using CT, AAAs with a diameter >50 mm were included in this study. Concurrently, we selected 100 healthy volunteers in whom AAA was excluded through CT examination. Patients with the following complications were excluded: Ehlers-Danlos syndrome; Marfan syndrome; and any other known vascular disorders, infections, autoimmune diseases, malignancies, psychosis, sleep disorders, those currently undergoing radiation therapy, shift workers, and patients with jet lag syndrome. In addition, patients who were currently taking immunosuppressants, sedatives, antiepileptic drugs, tricyclic antidepressants, or any drug that affects melatonin metabolism were excluded. This study was conducted in accordance with recommendations outlined in research ethics committee guidelines of The First Hospital of China Medical University. The protocol was approved by the Ethics Committee of The First Hospital of China Medical University. In accordance with the Declaration of Helsinki, all subjects provided written informed consent prior to participation in the study.

### Blood Samples

In this study, we collected blood samples at midnight under weak light (<100 lux) conditions, to ensure that melatonin secretion was not affected. All blood samples were stored at −80°C.

Serum melatonin and TNF-α levels were assessed in triplicate using a human melatonin enzyme-linked immunosorbent assay (ELISA) kit (Cusabio, China) and a human TNF-α ELISA kit (R&D Systems, USA), respectively. The serum total antioxidant status (TAS) was measured using a T-AOC (ABTS) kit (Beyotime, China), with a detection range of 0–3 mmol/L.

### Animal Modeling

Male Sprague-Dawley (SD) rats (300–320 g), provided by the Animal Experiment Department of China Medical University, were used in this experiment. All experiments involving rats were approved by the China Medical University Institutional Animal Care and Use Committee. All animal experiments complied with the China Medical University Guide for the Care and Use of Laboratory Animals. Rats were housed in a 12:12 h light:dark cycle at a temperature of 22 ± 2°C and a humidity of 65–70%. Sufficient drinking water and food were provided. During the modeling process, rats were anesthetized using isoflurane, the abdominal aorta was exposed *in situ*, and the lower abdominal aortic segment was dissociated. If necessary, adjacent branches were ligated. The abdominal aortic diameter was measured before perfusion. An incision was made at the branch of the iliac artery, a PE-10 tube was placed through the incision into the abdominal aorta, and the aorta was perfused for 10 min with porcine pancreatic elastase (2 U/ml in saline, E1250, Sigma-Aldrich, USA). The control group was treated with elastase that had been inactivated at 100°C for 20 min. Once the perfusion was complete, the catheter was withdrawn, and the vessel was sutured at the iliac artery incision with a 10-0 vascular suture. The abdominal incision was sutured with a 4-0 suture, and the rats were transferred to a clean room to recover. Fourteen days after surgery, the abdomen was exposed to visualize the abdominal aorta, and the abdominal aortic diameter was measured before sacrifice. AAA was defined as an expansion greater than 50%.

### Animal Experiment Design

The purpose of this experiment was to investigate the possible mechanisms underlying AAA development and to identify potential therapeutic targets. We applied the intraluminal elastase perfusion AAA model as our experimental model. To detect the mechanism underlying AAA development, rats were randomly assigned to either the heat-inactivated elastase group (*n* = 25) or the activated elastase group (*n* = 25). Western blotting (WB) and immunofluorescence staining were used to detect the levels of VSMC phenotype-related proteins, AKT-mTOR signaling pathway proteins, and autophagy-related proteins. In addition, we divided AAA rats into three groups, to study the effects of melatonin on nicotine-related AAA and to explore the possible underlying mechanisms: (1) Con (AAA model; *n* = 25), (2) Nic (*n* = 25), and (3) Nic + Mel (*n* = 25). The Nic group received nicotine (5 mg/kg/d) daily for 14 days *via* a tail vein injection, while the melatonin group received daily intraperitoneal injections of melatonin (20 mg/kg/d) for 14 days, as described in our previous study (Li et al., 2017). The control group received the vehicle. We evaluated vascular morphology and function using microscopy, hematoxylin and eosin (H&E) staining, and Verhoeff-Van Gieson (VVG) staining (Li et al., 2017). The effects of melatonin on VSMC phenotypic switching, AKT-mTOR signaling, autophagy, oxidative stress, and inflammation were evaluated using WB and immunofluorescence staining. All experiments described in this article were double-blind.

### Western Blot Analysis

Briefly, protein was extracted from the aorta using radioimmunoprecipitation assay (RIPA) buffer (Beyotime, China) and quantified using a BCA Protein Kit (CWBio, China). Proteins were separated using sodium dodecyl sulfate–polyacrylamide gel electrophoresis (SDS-PAGE) and transferred to polyvinylidene fluoride (PVDF) membranes. The PVDF membranes were blocked with 1% bovine serum albumin (BSA) for 1 h, and then incubated with primary antibodies against p-AKT (Cell Signaling Technology, USA), AKT (Cell Signaling Technology, USA), p-mTOR (Cell Signaling Technology, USA), mTOR (Cell Signaling Technology, USA), p62 (Cell Signaling Technology, USA), LC3 (Sigma-Aldrich, USA), SM22α (Abcam, UK), α-SMA (Abcam, UK), myocardin (Abcam, UK), OPN (Abcam, UK), matrix metalloproteinase-2 (MMP-2; Novus, USA), matrix metalloproteinase-9 (MMP-9; Abcam, UK), p65 (Cell Signaling Technology, USA), TNF-α (Abcam, UK), and β-actin (Abcam, UK), overnight at 4°C. Next, membranes were washed three times with tris buffered saline tween (TBST) and incubated for 1 h with secondary antibodies (Abcam, UK). Proteins were detected using enhanced chemiluminescent reagents (Thermo Fisher Scientific, USA) and quantified using ImageJ software (NIH, USA).

### Blood Pressure Measurement

Blood pressure of the rats was determined using a non-invasive blood pressure analysis system (Visitech Systems, Inc., USA). For blood pressure monitor adaptation, each rat underwent daily blood pressure measurement training at a fixed time. After 14 days of training, the monitor was used to obtain stable systolic pressure, diastolic pressure, and heart rate.

### Immunofluorescence Staining

Abdominal aortas were frozen and embedded in optimal cutting temperature (OCT) compound. Tissue sections (5 μm) were incubated with primary antibodies against α-SMA (Abcam, UK), OPN (Abcam, UK), MMP-2 (Novus, USA), MMP-9 (Abcam, UK), and iNOS (Abcam, UK) in a humidified chamber overnight at 4°C. Next, sections were washed three times with TBST and incubated with Alexa Fluor 594-conjugated secondary antibodies (Abcam, USA). Nuclei were stained with 4',6-diamidino-2-phenylindole (DAPI). Immunofluorescence was visualized using an Olympus fluorescence microscope.

### Animal Blood Samples

In this study, we collected blood samples from the Con, Nic, and Nic + Mel groups. All blood samples were stored at −80°C. Serum FSH, luteinizing hormone, and testosterone levels were assessed in triplicate using a rat FSH ELISA kit (CCC, USA), a rat LH ELISA kit (CCC, USA), and a rat testosterone ELISA kit (CCC, USA), respectively.

### Statistical Analysis

All graphs were constructed using GraphPad Prism software (GraphPad Software Inc.). The data are presented as means ± SEMs. Differences between two groups were analyzed using the two-tailed Student’s *t*-test, while comparisons between multiple groups were performed using one-way ANOVA, followed by the Bonferroni *post hoc* test. A value of *p* < 0.05 was considered statistically significant. All experiments were repeated at least three times.

## Results

### Melatonin Levels in Patients With AAA

Two hundred people were enrolled in this study: 100 people each in the AAA group and the normal control group. Table 1 shows basic information for both patients with AAA and control participants. No significant differences in sex, age, hypertension status, smoking status, presence of dyslipidemia, chronic obstructive pulmonary disease (COPD), IHD, or noninsulin-dependent diabetes mellitus (NIDDM) were observed between the two groups (Table 1).

**Table 1:** Baseline characteristics of patients with AAA and control subjects.

Variables	AAA (*n* = 100)	Controls (*n* = 100)	*p*-value
Men/women (*n*)	80/20	81/19	0.858
Age (years)	59.6 ± 3.8	60.4 ± 5.0	0.247
Hypertension (%)	57	49	0.257
Smoker (%)	61	52	0.199
Dyslipidemia (%)	37	31	0.370
COPD (%)	5	4	0.733
IHD (%)	12	15	0.535
NIDDM (%)	7	8	0.788

COPD, chronic obstructive pulmonary disease; IHD, ischemic heart disease; NIDDM, noninsulin-dependent diabetes mellitus.

**Figure 1:**
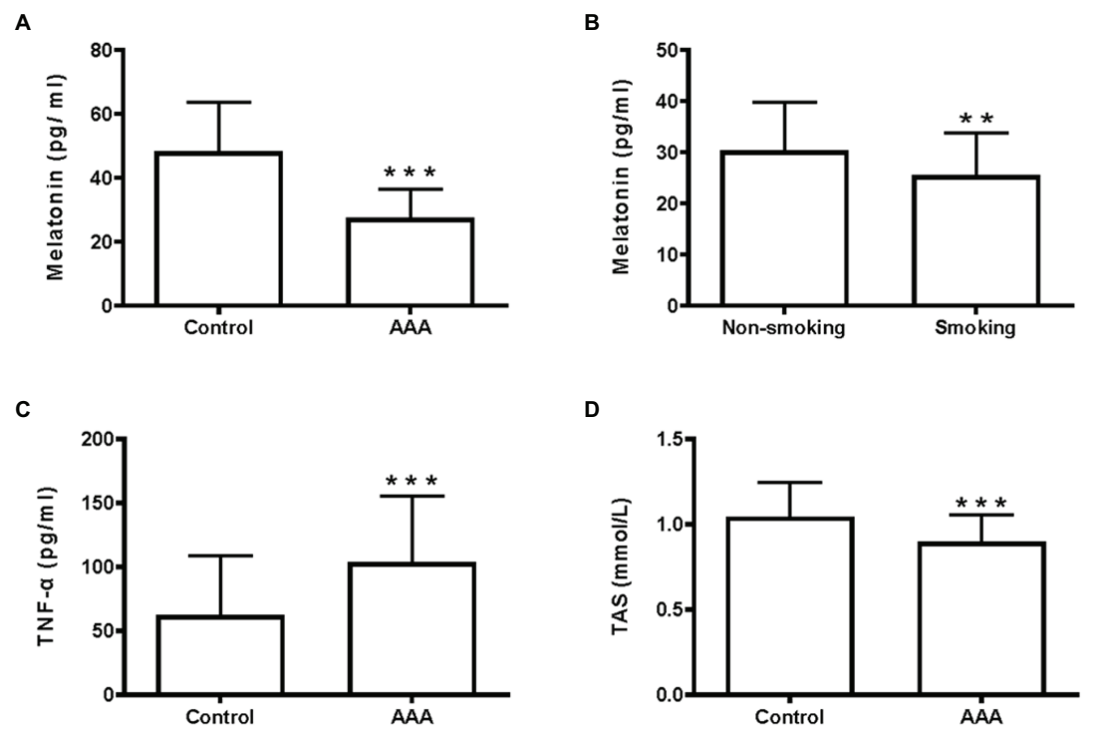
Melatonin, TNF-α, and total antioxidant status (TAS) levels in the clinical samples. **(A)** Serum melatonin levels, ^***^p < 0.001 compared with the control group. **(B)** Melatonin levels in the smoking and non-smoking subgroups of patients with abdominal aortic aneurysm (AAA), ^**^p < 0.01 compared with the non-smoking group. **(C)** Serum TNF-α levels, ^***^p < 0.001 compared with the control group. **(D)** Serum TAS levels, ^***^p < 0.001 compared with the control group.

Melatonin levels in the AAA group were significantly lower than those in the control group (*p* < 0.001; Figure 1A). Significantly lower melatonin levels were observed in the smoking group of patients with AAA, compared to the non-smoking group (*p* < 0.01; Figure 1B).

**Figure 2:**
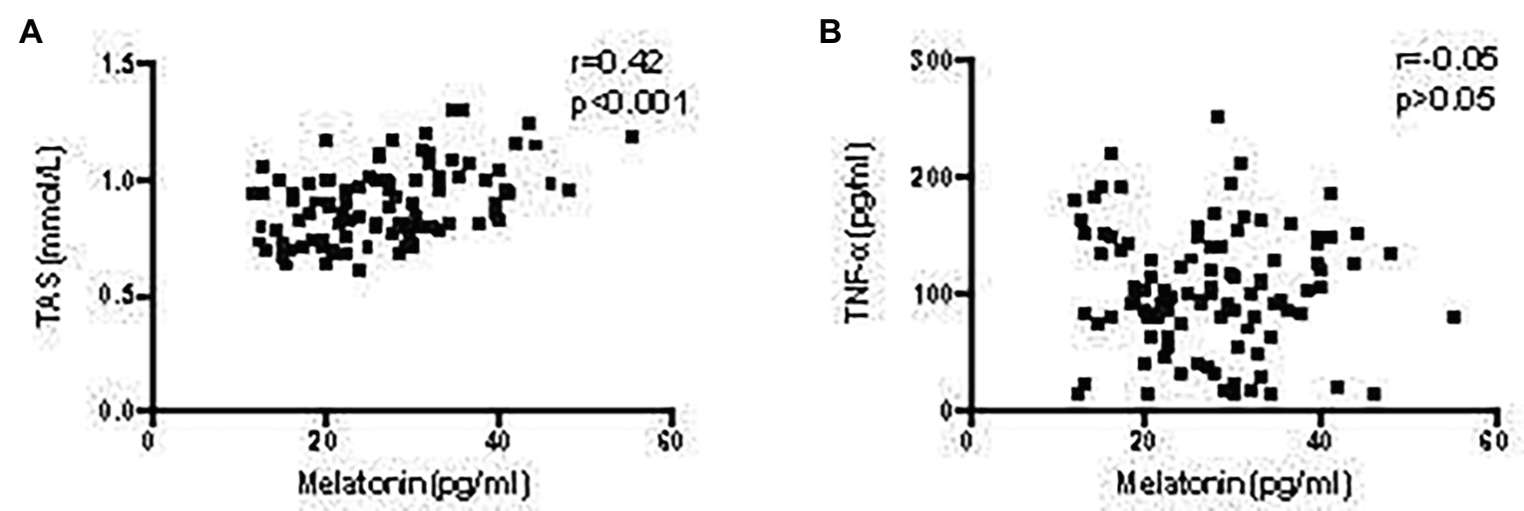
Correlation of melatonin levels with TAS and TNF-α levels in patients with AAA. **(A)** Correlation between melatonin and TAS levels. **(B)** Correlation between melatonin and TNF-α levels.

As shown in our previous study, the inflammatory environment of the arterial wall promotes AAA development (Li et al., 2017). In the present study, serum TNF-α levels were significantly higher in patients with AAA, than in the control group (*p* < 0.001; Figure 1C). Furthermore, a lower serum TAS level was observed in patients with AAA, compared to the control group (*p* < 0.001; Figure 1D). These results identified a positive correlation between melatonin levels and TAS (*r* = 0.42, *p* < 0.001; Figure 2A), while no relationship was observed between melatonin and TNF-α levels (Figure 2B).

### AAA Formation in the AAA Rat Model

**Figure 3:**
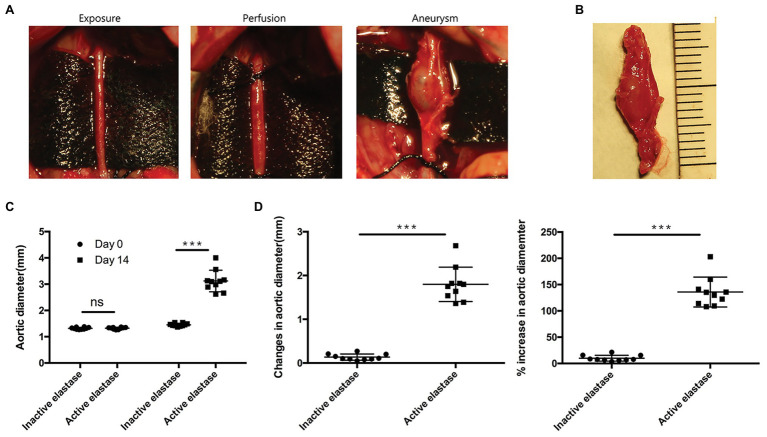
Elastase perfusion of the infrarenal abdominal aorta resulted in AAA formation (*n* = 10). **(A)** Elastase perfusion of the infrarenal abdominal aorta before (day 0) and after perfusion (day 14). **(B)** An AAA observed 14 days after elastase perfusion. **(C)** Abdominal aortic diameter measured on days 0 and 14. ns, no significant difference; ^***^p < 0.001. **(D)** Changes (left panel) and the % increase (right panel) in the abdominal aortic diameter 14 days after perfusion, ^***^p < 0.001.

To explore the mechanisms underlying AAA formation, we first established an AAA rat model. Noticeable AAAs were formed on the 14th day after perfusion (Figures 3A,B). No significant difference in pre-perfusion baseline infrarenal aortic diameter was observed between the two groups (*p* > 0.05; Figure 3C). Moreover, we measured increases in aortic diameter and expansion of the abdominal aorta. These values were significantly higher in activated elastase-perfused rats compared to heat-inactivated elastase-perfused rats (*p* < 0.001; Figure 3D).

### Melatonin Attenuated Aortic Expansion in the Nicotine-Related AAA Model

**Figure 4:**
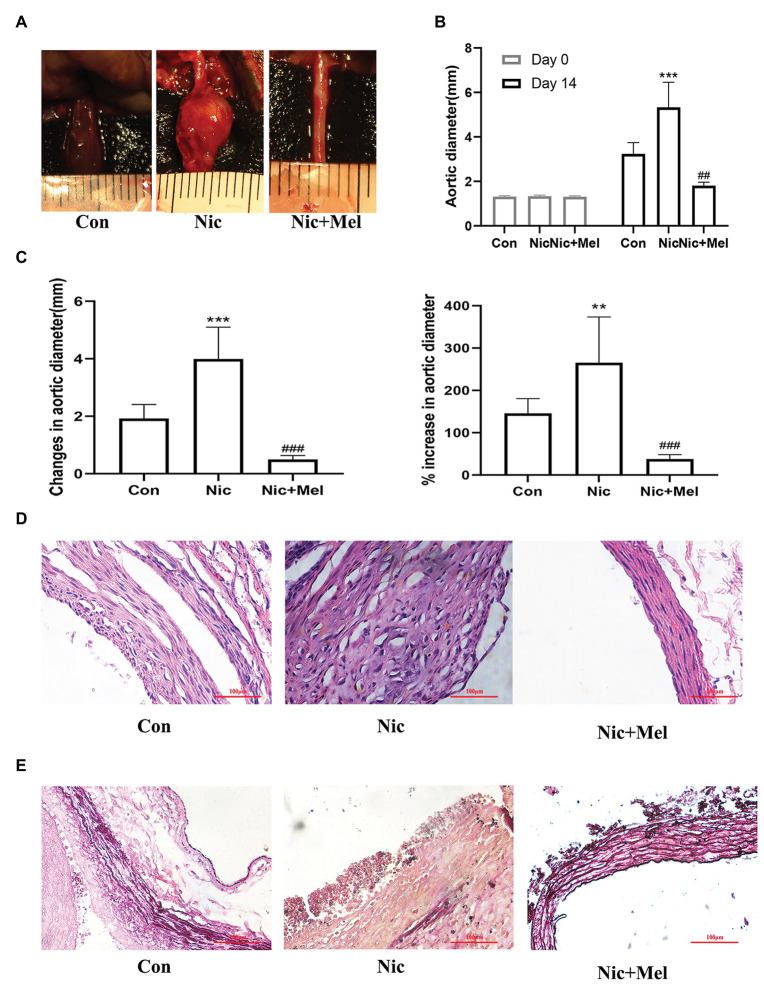
Nicotine accelerated AAA expansion, while melatonin attenuated AAA expansion (*n* = 10). **(A)** Effects of nicotine and melatonin on AAA formation. **(B)** Abdominal aortic diameter measured on days 0 and 14, ^***^p < 0.001 and ^###^*p* < 0.001. **(C)** Changes (left panel) and the % increase (right panel) in the abdominal aortic diameter, ^**^p < 0.01, ^***^p < 0.001 and ^###^*p* < 0.001. **(D)** H&E staining of tissues from the Con, Nic, and Nic + Mel groups. **(E)** Verhoeff-Van Gieson (VVG) staining of tissues from the Con, Nic, and Nic + Mel groups.

Abdominal aortic diameter was significantly larger in the Nic group than in the Con group, while in the Nic + Mel group, abdominal aortic diameter was smaller than in the Nic group (*p* < 0.001; Figures 4A–C). Compared to the arterial wall in the Con group, the arterial wall structure in the Nic group was severely damaged and infiltrated with a greater number of inflammatory cells, as evidenced through H&E staining. However, compared to the Nic group, the arterial wall structure of the Nic + Mel group was clear and complete, with no significant inflammatory cell infiltration (Figure 4D). VVG staining showed a complete disruption, and near absence, of elastic fibers in the Nic group, compared to the Con group. The elastic fiber structure was restored in the Nic + Mel group (Figure 4E).

### Melatonin Reduced Blood Pressure in the Nicotine-Related AAA Model

**Figure 5:**
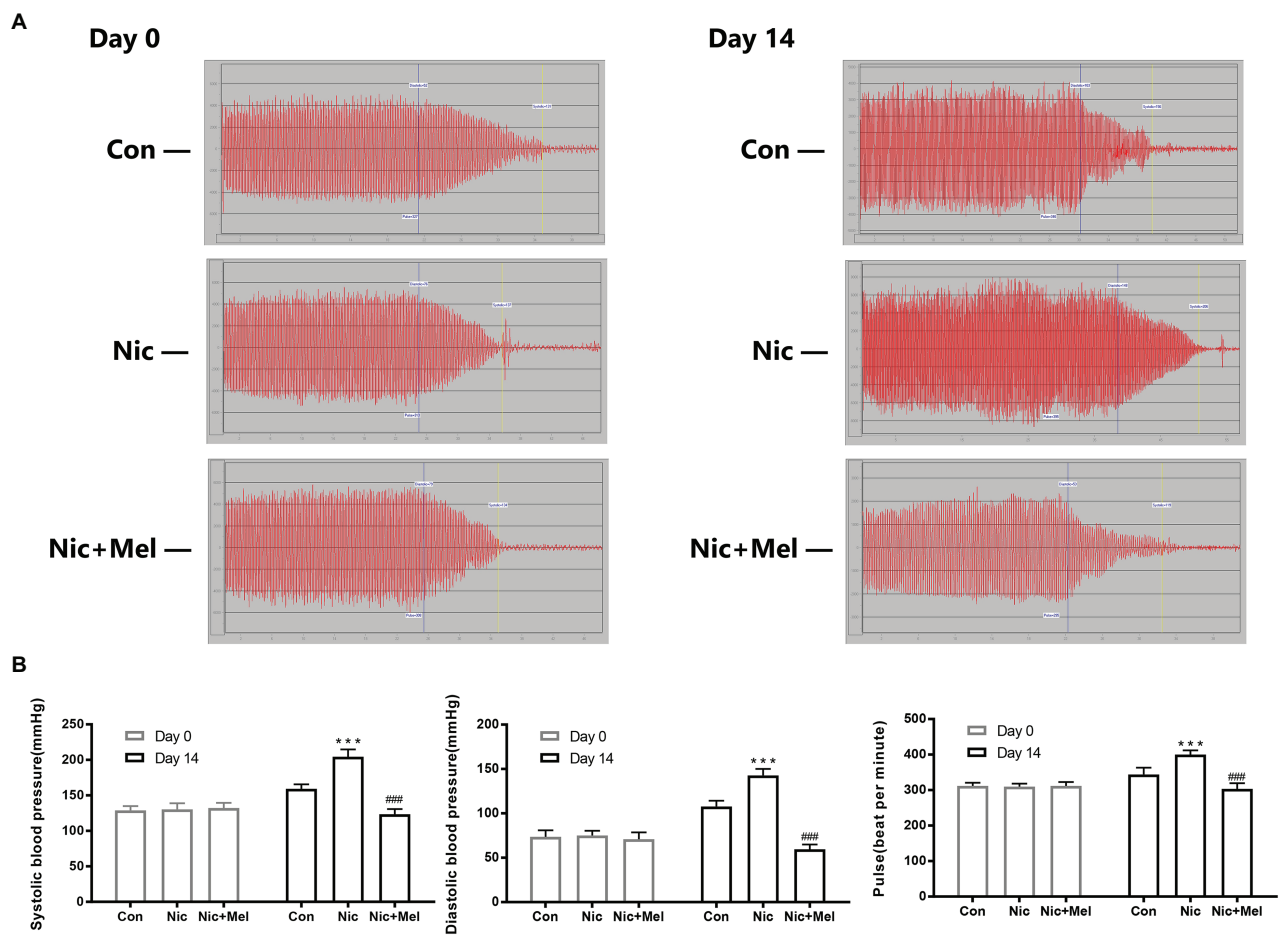
Non-invasive blood pressure monitoring of systolic blood pressure, diastolic blood pressure, and pulse rate. **(A)** Waveforms of the systolic blood pressure, diastolic blood pressure, and pulse rate (days 0 and 14). **(B)** Systolic blood pressure, diastolic blood pressure, and pulse rate in the Con, Nic, and Nic + Mel groups on days 0 and 14, ^***^p < 0.001 and ^###^*p* < 0.001.

To further investigate the effects of nicotine and melatonin on blood pressure, we measured systolic blood pressure, diastolic blood pressure, and pulse rate (Figure 5A). On day 14, systolic blood pressure, diastolic blood pressure, and pulse rate were significantly higher in the Nic group than in the Con group (*p* < 0.001; Figure 5B). However, the Nic + Mel group presented with significantly lower systolic blood pressure, diastolic blood pressure, and pulse rate, compared to the Nic group (*p* < 0.001; Figure 5B). Based on these data, melatonin treatment reduced blood pressure in rats with nicotine-related AAA.

### AKT-mTOR Signaling Was Activated, Autophagy Function Was Impaired, and the VSMC Phenotype Was Transformed From the Contractile to the Synthetic Phenotype During AAA Formation

**Figure 6:**
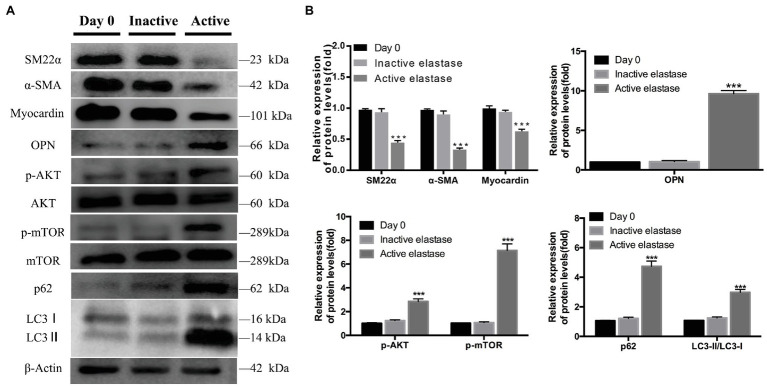
Phenotypic switching of vascular smooth muscle cells (VSMCs), activation of the AKT-mTOR pathway, and autophagy dysfunction during AAA formation. **(A)** Immunoblots showing the levels of phosphorylated AKT and total AKT, phosphorylated mTOR and total mTOR, p62, LC3I, LC3II, SM22α, α-SMA, myocardin, and OPN in the activated elastase-perfused group and heat-inactivated elastase-perfused group on day 0. **(B)** Total AKT, mTOR, and β-actin were used as the standards, ^***^p < 0.001 for the comparison of the heat-inactivated elastase-perfused group with the activated elastase-perfused group.

On day 14, significantly lower levels of contractile proteins were observed in elastase-perfused aortas, than in control aortas, (*p* < 0.001). In contrast, OPN was expressed at significantly higher levels on day 14 in elastase-perfused aortas than in heat-inactivated elastase-perfused aortas (*p* < 0.001; Figures 6A,B). Significantly higher levels of p-AKT and p-mTOR were observed in the activated elastase-perfused group than in the heat-inactivated elastase-perfused group (Figures 6A,B). The LC3II/LC3I ratio was significantly increased in the activated elastase-perfused group, indicating promotion of LC3I conversion to autophagosome-associated LC3II. In addition, we were surprised to observe substantial accumulation of p62, indicating autophagy inhibition (Figures 6A,B).

### Melatonin Inhibited AKT-mTOR Signaling, Improved Autophagy Dysfunction, and Restored the VSMC Phenotype in the Nicotine-Related AAA Model

**Figure 7:**
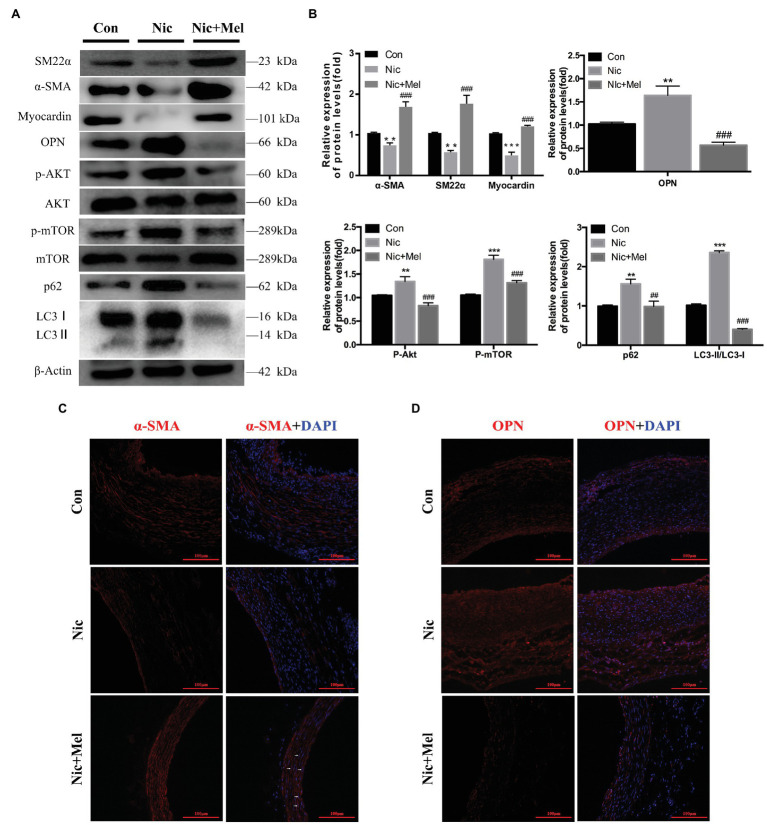
Nicotine further promoted the phenotypic switching of VSMCs, activation of AKT-mTOR pathway, and aggravation of autophagic injury, while melatonin significantly reversed the effects of nicotine. **(A)** Immunoblots showing the levels of phosphorylated AKT and total AKT, phosphorylated mTOR and total mTOR, p62, LC3I, LC3II, SM22α, α-SMA, myocardin, and OPN in the Con, Nic, and Nic + Mel groups. **(B)** Total AKT, mTOR, and β-actin were used as the standards, ^**^p < 0.01 and ^***^p < 0.001 for the comparison of the Nic group with the Con group, ^##^*p* < 0.01 and ^###^*p* < 0.001 for the comparison of the Nic + Mel group with the Nic group. **(C)** Images of immunofluorescence staining for α-SMA (red) and 4',6-diamidino-2-phenylindole (DAPI; blue) in tissues from the Con, Nic, and Nic + Mel groups. **(D)** Images of immunofluorescence staining for OPN (red) and DAPI (blue) in tissues from the Con, Nic, and Nic + Mel groups.

Establishment of nicotine-related AAA reduced contractile protein expression in VSMCs and increased expression of the synthetic phenotype marker OPN, indicating that nicotine promotes VSMC phenotypic switching and vascular injury (Figures 7A,B). As expected, melatonin treatment restored expression of VSMC phenotypic markers (Figures 7A,B). Moreover, significantly higher levels of p-AKT and p-mTOR were observed in the Nic group than in the Con group. In contrast, melatonin inhibited activation of the AKT-mTOR signaling pathway (Figures 7A,B). In addition, a significantly higher LC3II/LC3I ratio was observed in the Nic group, compared to the Con group, and expression of autophagy substrate p62 significantly increased; thus indicating further autophagy inhibition (Figures 7A,B). In the Nic + Mel group, the LC3II/LC3I ratio was reduced, and p62 expression decreased, indicating that the autophagy flux was stabilized. Immunofluorescence staining revealed lower α-SMA expression in the Nic group, compared to the Con group; however, following melatonin treatment, α-SMA expression was restored, and cells exhibited a spindle morphology (shown by the white arrows in Figure 7C). Immunofluorescence staining revealed higher OPN expression in the Nic group, compared to the Con group, while melatonin treatment significantly decreased OPN expression (Figure 7D).

### Melatonin Reduced Inflammation and Oxidative Stress Levels and Restored Gonadal Hormone Levels in the Nicotine-Related AAA Model

**Figure 8:**
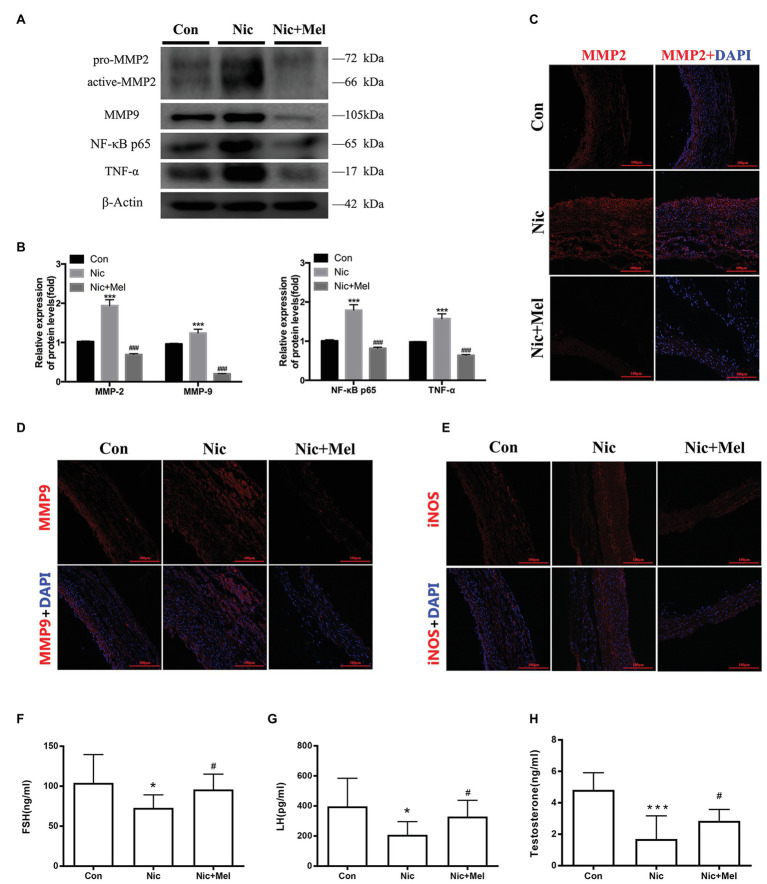
Nicotine further promoted inflammation, increased oxidative stress levels, and decreased gonadal hormone levels, while melatonin significantly reversed the effects of nicotine. **(A)** Immunoblots showing the levels of MMP-2, MMP-9, P65, and TNF-α in the Con, Nic, and Nic + Mel groups. **(B)** β-actin was used as the standard, ^***^p < 0.001 for the comparison of the Nic group with the Con group and ^###^*p* < 0.001 for the comparison of the Nic + Mel group with the Nic group. **(C)** Images of immunofluorescence staining for MMP-2 (red) and DAPI (blue) in tissues from the Con, Nic, and Nic + Mel groups. **(D)** Images of immunofluorescence staining for MMP-9 (red) and DAPI (blue) in tissues from the Con, Nic, and Nic + Mel groups. **(E)** Images of immunofluorescence staining for iNOS (red) and DAPI (blue) in tissues from the Con, Nic, and Nic + Mel groups. **(F)** Serum FSH levels, ^*^p < 0.05 for the comparison of the Nic group with the Con group and ^#^*p* < 0.05 for the comparison of the Nic + Mel group with the Nic group. **(G)** Serum LH levels, ^*^p < 0.05 for the comparison of the Nic group with the Con group and ^#^*p* < 0.05 for the comparison of the Nic + Mel group with the Nic group. **(H)** Serum testosterone levels, ^***^p < 0.05 for the comparison of the Nic group with the Con group and ^#^*p* < 0.05 for the comparison of the Nic + Mel group with the Nic group.

Finally, we detected inflammation and oxidative stress levels in the abdominal aorta using WB and immunofluorescence staining. WB revealed significantly higher levels of MMP-2, MMP-9, NF-κB, P65, and TNF-α in the Nic group, compared to the Con group, while melatonin reduced the level of each of these proteins (Figures 8A,B). To further study the relationships between AAA and both inflammation and oxidative stress, we evaluated MMP-2, MMP-9, and iNOS expressions using immunofluorescence staining. Immunofluorescence staining revealed higher expressions of MMP-2, MMP-9, and iNOS in the Nic group, compared to the control group, while all were expressed at lower levels in the Nic + Mel group, compared to the Nic group (Figures 8C–E). Thus, nicotine significantly increased inflammation and oxidative stress levels in the abdominal aorta, while melatonin reduced the effects of nicotine. ELISA results showed lower levels of FSH, LH, and testosterone in the Nic group, compared to the Con group, while melatonin increased the level of each of these hormones (Figures 8F–H).

### Melatonin Lost Its Protective Effects on AAA Formation When Bafilomycin A1 Was Administered to Inhibit Autophagy

**Figure 9:**
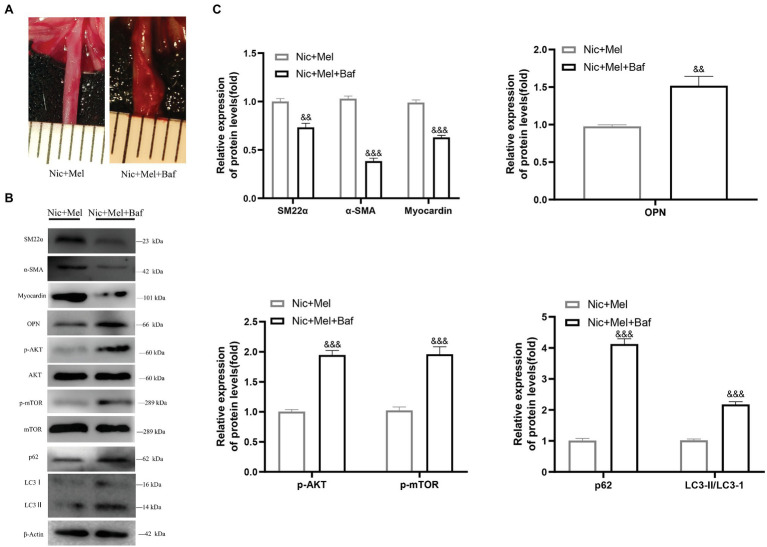
Melatonin lost its protective effects on AAA formation following the administration of bafilomycin A1 to inhibit autophagy. **(A)** The effect of bafilomycin A1 on AAA formation. **(B)** Immunoblots showing the levels of phosphorylated AKT and total AKT, phosphorylated mTOR and total mTOR, SM22α, α-SMA, myocardin, OPN, LC3I, LC3II, and p62 in the Nic + Mel group and Nic + Mel + Baf group. **(C)** Total AKT, mTOR, and β-actin were used as the standards, ^&&^*p* < 0.01 and ^&&&^*p* < 0.001 for the comparison of the Nic + Mel + Baf group with the Nic + Mel group.

To further explore the mechanism of autophagy in melatonin-treated nicotine-related AAA, we divided SD rats into two groups, namely, the melatonin-treated nicotine-related AAA group (Nic + Mel) and the bafilomycin A1‐ and melatonin-treated group (Nic + Mel + Baf; *n* = 10). Previous results showed that aneurysms had not formed in Nic + Mel group rats; however, they had formed in Nic + Mel + Baf rats (Figure 9A). We found that bafilomycin A1-treated AAAs reduced contractile protein expression in VSMCs and increased expression of the synthetic phenotype marker OPN, indicating that bafilomycin A1 promotes VSMC phenotypic switching and vascular injury (Figures 9B,C). Next, we found that p-AKT and p-mTOR expression was significantly higher in the Nic + Mel + Baf group, compared to the Nic + Mel group (Figures 9B,C). In addition, we detected LC3 protein levels. A significantly higher LC3II/LC3I ratio was observed in the Nic + Mel + Baf group, compared to the Nic + Mel group, while expression of autophagy substrate p62 also significantly increased, indicating autophagy inhibition (Figures 9B,C). Based on these results, bafilomycin A1 reversed the protective effects of melatonin on AAA formation. Furthermore, it promoted VSMC conversion from the contractile phenotype to the synthetic phenotype and aggravated autophagy dysfunction. In conclusion, melatonin lost its protective effects on AAA formation following bafilomycin A1 administration and autophagy inhibition.

## Discussion

Our study is the first to show that melatonin plays a novel protective role in nicotine-related AAA. Based on our results, melatonin attenuated nicotine-related AAA development by restoring the VSMC phenotype, inhibiting the AKT-mTOR pathway, ameliorating autophagy dysfunction, and promoting anti-inflammatory and antioxidant activities. These findings suggest a potential therapeutic role for melatonin in the clinical treatment of AAA.

In our experiment, lower serum melatonin levels were observed in patients with AAA than in control participants; this pattern was similar to that observed for other cardiovascular diseases (Dominguez-Rodriguez et al., 2008). Moreover, serum melatonin levels in smokers with AAA were lower than in non-smoking patients with AAA, consistent with previous reports which indicated that smoking reduces serum melatonin levels (Ozguner et al., 2005). In the human body, melatonin is an important component of serum antioxidant capacity. As the level of melatonin positively correlated with TAS, this indicates that, to some extent, melatonin levels reflect the antioxidant capability of patients with AAA. This finding identifies melatonin supplementation as a strategy for treating patients with AAA; furthermore, melatonin may be a biomarker for predicting AAA.

Ample evidence has shown that smoking is perhaps the unique modifiable risk factor for cardiovascular diseases, including AAA, and that nicotine, the main component of smoke, causes oxidative stress and interstitial fibrosis, which contributes to smoking-induced heart diseases (Hu and Ren, 2014, 2016; Kilic et al., 2018). Furthermore, as nicotine has been proven to exert an important effect on VSMC function (Cucina et al., 2008; Vazquez-Padron et al., 2010; Wang et al., 2012; Yoshiyama et al., 2014; Liang et al., 2017), we chose to focus on nicotine in this study. In addition, this model enabled us to observe vascular morphological changes, such as lumen expansion, in a timely manner. Based on accumulating evidence from recent studies, melatonin exerts therapeutic effects on ischemia-reperfusion injury, hypertension, atherosclerosis, and pulmonary arterial hypertension (Baker and Kimpinski, 2018; Wang et al., 2018a). As shown in a study by Astorga et al. (2018), melatonin reduces both oxidative stress and pathological remodeling, while improving vascular structure and function (Astorga et al., 2018). In addition, melatonin inhibits the expression of NF-κB and exerts anti-inflammatory effects (Jin et al., 2014; Prado et al., 2018). Furthermore, melatonin exerts a protective effect on smoke-induced restenosis (Yang et al., 2014). While nicotine accelerates abdominal aortic expansion, melatonin attenuates this expansion rate, indicating that melatonin may exert protective effects on nicotine-related AAA. Additionally, nicotine induced significant changes in the structure and function of the arterial wall, endothelial injury, and abnormal VSMC proliferation in the present study. Nicotine also increased inflammatory cell infiltration, vascular fibrosis, and the degree of aortic elastic fiber rupture. In contrast, melatonin reduced inflammation and improved vascular structure and function. These results are consistent with clinical reports and suggest that smoking cessation is beneficial to preventing AAA rupture.

VSMCs are the main cells constituting vascular wall tissue, also maintaining vascular elasticity. VSMC dysfunction is the pathological basis of vascular disease. When VSMCs are activated by multiple cytokines, they transform from the contractile phenotype to a phenotype with proliferative and migratory functions, which closely relates to the cardiovascular disease process (Sun et al., 2017). Contractile protein expression restoration contributes to the vascular remodeling process and improves arterial structure (Ren et al., 2017; Lino Cardenas et al., 2018). In the present study, during AAA progression, the VSMC phenotype was transformed and resulted in the arterial wall losing its elasticity, thus contributing to expansion. Here, melatonin restored the VSMC phenotype in nicotine-related AAA, suggesting that the reversal of VSMC phenotypic switching is a key target for AAA treatment.

Many factors may affect VSMC phenotypic switching. As mentioned above, activation of the mTOR pathway is related to the contractile phenotype of VSMCs during the development of AAA. Several recent studies have confirmed that AKT signaling pathways are closely related to vascular diseases. For example, activation of the AKT signaling pathway increases MMP-2 expression and aggravates atherosclerotic plaque formation (Lee et al., 2008; Yao et al., 2018), while its downregulation may inhibit abnormal VSMC proliferation and migration (Lee et al., 2018). However, no relevant study has analyzed the relationship between the AKT-mTOR pathway and AAA. The AKT-mTOR pathway was significantly activated in nicotine-related AAA, with melatonin downregulating this pathway in the present study. This suggests that melatonin may restore the VSMC phenotype through inhibition of the AKT-mTOR pathway.

Based on our results, melatonin treatment reduced blood pressure in the nicotine-related AAA model. As accumulating evidence has shown that melatonin affects blood pressure, a systematic review and meta-analysis, including five randomized controlled trials (RCTs), identified a significant reduction in systolic blood pressure and diastolic blood pressure after supplementation with melatonin, compared with a control treatment (Hadi et al., 2019). The proposed mechanisms underpinning melatonin’s effects on blood pressure include: anti-inflammatory action *via* inhibition of the cyclooxygenase-2 (COX-2) enzyme, scavenging of free radicals, activation of antioxidant defense enzymes, an increase in nitric oxide (NO) production, and reduction of great artery resistance to blood flow. These effects lead to protection against vascular endothelial damage and vasoconstriction. According to the Society for Vascular Surgery practice guidelines on the care of patients with AAA, blood pressure is a potential risk factor for AAA, and men with AAA should strictly control their blood pressure. As shown in the study by Kobeissi et al. (2019), hypertension increases the risk of developing AAA by 66% (Kobeissi et al., 2019). In addition, hypertension may accelerate the expansion rate of AAA and increase the risk of rupture. Currently, one of the most important therapeutic methods for preventing AAA rupture is decreasing blood pressure. In our present study, nicotine and melatonin increased and decreased blood pressure, respectively (Figure 5). Thus, the anti-hypertensive effect of melatonin may decrease both the expansion rate and risk of AAA rupture.

In addition, as autophagy has been reported to be closely related to vascular disease, we studied changes in the levels of autophagy-related proteins (Grootaert et al., 2018; Ou et al., 2018; Yang et al., 2018; Zhu and Du, 2018). Changes in autophagy-related protein levels have also been found to alter VSMC and vascular endothelial cell functions (Xu et al., 2017, 2018; Chen et al., 2018; Sung et al., 2018). Both the LC3II/LC3I ratio and p62 expression increased significantly in the Nic group, compared with the Con group, with melatonin inhibiting p62 expression. Based on the results of *in vivo* experiments, melatonin lost its protective effects on AAA following administration of bafilomycin A1 for autophagy inhibition. We usually monitor autophagy flux to evaluate the process of autophagy. A substantial accumulation of p62 indicates that autophagy is impaired. It is also an important source of inflammation and oxidative stress in the body, which causes cellular dysfunction (Moscat and Diaz-Meco, 2009). Therefore, melatonin restored the VSMC phenotype through amelioration of autophagy dysfunction.

Inflammation and oxidative stress are considered important causes of AAA development, and inflammatory cell infiltration in the aortic wall is a classic pathological change in AAA (Windsor et al., 2018). In the present study, nicotine exacerbated inflammatory cell infiltration in AAA, while melatonin reduced the inflammatory response. Additionally, MMP-2, MMP-9, and TNF-α expression was significantly increased in the Nic group, compared with the Con group, while melatonin inhibited the expression of each of these proteins. Inflammatory cells secrete MMPs, which degrade the extracellular matrix. In addition, inflammatory cytokine secretion stimulates VSMCs to secrete MMPs, thus promoting inflammatory cell infiltration and continued destruction of the arterial wall. Here, we specifically examined the expression of NF-κB p65, which is widely associated with inflammatory factors, chemokines, oxidative stress-related enzymes, and adhesion factors. Furthermore, smoking has been reported to cause vascular inflammation through NF-κB p65. In the present study, melatonin reduced NF-κB p65 expression, which may be the key mechanism underlying the effects of melatonin on the treatment of nicotine-related AAA.

Melatonin is predominately synthesized and secreted by the pineal gland. A decrease in melatonin levels has been shown to correlate with the progression of Tanner stages, which contributes to emerging concern relating to the possibility that exogenous melatonin supplementation may affect the sex hormones. Therefore, we measured the levels of FSH, LH, and testosterone. Nicotine decreased the levels of these hormones, while melatonin restored their levels. According to Fahimeh et al. melatonin ameliorates testicular damage induced by nicotine in mice, increases serum levels of testosterone and LH, and improves sperm parameters, including morphology, motility, and counts (Mohammadghasemi and Jahromi, 2018). These results were similar to the findings of the current study. Hence, melatonin ameliorated the reproductive damage caused by nicotine in the nicotine-related AAA model.

Faulty immune regulation was also recently shown to contribute to aneurysm formation and growth (Wang et al., 2018b). The aorta wall loses its medial immune-privileged status, providing a permissive environment for immune cell infiltration and cytokine secretion, which may promote AAA (Cuffy et al., 2007). Previous studies have shown that melatonin is an endogenous antioxidant and anti-inflammatory molecule. Moreover, melatonin was reported to exert powerful immunomodulatory effects (Wongsena et al., 2018), with its immunomodulatory properties and effects on the immune cell response vital for protecting the abdominal aorta and VSMCs. In our study, we did not assess the effect of melatonin on immune cells; however, according to published evidence, the immunomodulatory effects of melatonin, particularly the contribution to medial immune privilege, are an additional mechanism for preventing AAA formation. Future studies are needed to clarify the effects of melatonin on immune cells during AAA formation. Although high doses of melatonin have been reported to be safe in clinical trials (Gitto et al., 2009), we must investigate the potential for off-target effects on other cells, particularly macrophages and T cells, which are also critical for AAA formation. Dose-response studies must be performed in the future to evaluate the most suitable dose to protect the abdominal aorta.

Melatonin is a circadian endocrine molecule produced in the pineal gland. In recent years, accumulating evidence has suggested profound cardiovascular effects of melatonin (Zhou et al., 2018). The mechanism of cardiovascular disease is very complicated and remains unclear; for example, overconsumption of oxygen, intracellular calcium overload, cardiomyocyte death, microvascular perfusion defects, platelet hyperactivity, and other factors are proposed potential pathophysiological mechanisms (Lefaki et al., 2017; Liu et al., 2017; Sozen and Ozer, 2017; Steven et al., 2017). Through its powerful antioxidant and anti-inflammatory properties, melatonin was recently shown to combat mitochondrial impairment and cell death, suggesting its therapeutic potential in the cardiovascular field, including AAA. Consistent with our results, some studies observed a reduction of melatonin levels in patients, highlighting the necessity of melatonin supplements for reducing the risk of acute cardiovascular events (Takeda and Maemura, 2016; Romo-Nava et al., 2017). Therefore, further studies are needed to investigate the effects and mechanisms of melatonin on nicotine-related AAA.

**Figure 10:**
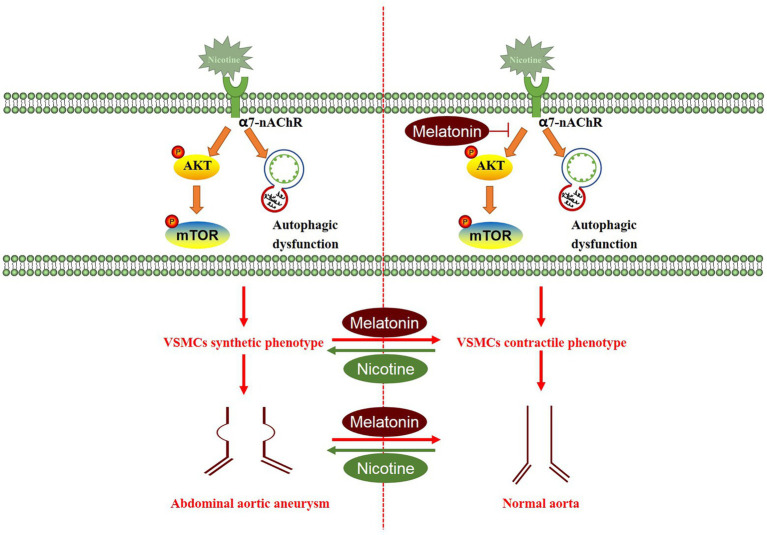
Proposed working model through which melatonin attenuates aortic expansion in the nicotine-related AAA model. Nicotine activated the AKT-mTOR signaling pathway and induced autophagy dysfunction, thus promoting the switch of the VSMC phenotype from the contractile phenotype to the synthetic phenotype. Melatonin reversed the effects of nicotine, thereby inhibiting aortic expansion.

Overall, we detected insufficient endogenous melatonin levels in patients with AAA, while smoking further reduced melatonin levels. Nicotine aggravated AAA-induced damage, which closely related to VSMC phenotype switching, from the contractile phenotype to the synthetic phenotype. Melatonin restored the VSMC phenotype by inhibiting the AKT-mTOR pathway, improving autophagy dysfunction, and reducing inflammation and oxidative stress levels, to ultimately attenuate the development of aneurysms. Melatonin exerted clear protective effects on nicotine-related AAA, highlighting its considerable potential as an AAA treatment (Figure 10). Melatonin, an endogenous human hormone, has a substantial safety advantage and it may be the potential drug of choice for AAA treatment in smokers. Further clinical trials are needed to evaluate its clinical effectiveness.

## Data Availability Statement

The datasets generated for this study are available on request to the corresponding author.

## Ethics Statement

The studies involving human participants were reviewed and approved by The Ethical Committee of The First Hospital of China Medical University. The patients/participants provided their written informed consent to participate in this study. The animal study was reviewed and approved by China Medical University Institutional Animal Care and Use Committee.

## Author Contributions

LD, SL, GL, LZ, and SX designed and performed the experiments and wrote the manuscript. LW, YJ, and WS helped LD to carry out experiments. YS and YL contributed to analysis and interpretation of the data and were responsible for critically revising the manuscript. All authors contributed to the article and approved the submitted version.

### Conflict of Interest

The authors declare that the research was conducted in the absence of any commercial or financial relationships that could be construed as a potential conflict of interest.
